# Women expert gamers: portrait of an understudied population

**DOI:** 10.3389/fpsyg.2025.1601425

**Published:** 2025-06-23

**Authors:** Roxanne Hébert-Ratté, Magali Dufour, Geneviève Martel-Brosseau, Ghassan El-Baalbaki

**Affiliations:** Department of Psychology, Université du Québec à Montréal, Montreal, QC, Canada

**Keywords:** women, competitive gaming, gaming expertise, self-esteem, wellbeing, gaming disorder, mental health, game genre

## Abstract

**Introduction:**

Very little is known about women who persist in competitive gaming environments and their unique characteristics. Thus, the current study aims to provide an initial portrait of these women.

**Methods:**

A subsample of 128 female gamers, selected from a larger internet survey, completed an online questionnaire assessing sociodemographic factors, gaming experience, motives to play, and mental health variables. Self-reported expert gamers were compared to casuals on these variables. Then, a backward logistic regression allowed to identify the best predictors of being an expert gamer.

**Results:**

Experts reported higher gaming involvement and higher levels of specific gaming motives. Experts also tended to engage in specific game types and to report more positive outcomes than casuals. Finally, gaming involvement, competition motive, self-esteem, and game genre were the best predictors of group membership.

**Discussion:**

This study highlighted several unique characteristics of female expert gamers, suggesting an important role of personal and environmental strengths, gaming involvement, and gender representation.

## Introduction

In the past two decades, the proportion of women involved in gaming has increased in many countries across the world (Basuroy, 2024; Clement, 2024a, 2024b, 2023c). On the other hand, involvement in competitive, skillful gaming and eSports has gained massive visibility in recent years, to the point where eSports is now a billion-dollar global industry (Clement, 2025d) with competitions and tournaments viewed by hundreds of millions of viewers (Clement, 2025c). Despite their presence being more widespread and normalized in gaming, women who make it at the top levels of competitive videogame play are rare. For example, among the top 10 eSports players by earnings, both in 2024 and January 2025, there was no woman (Clement, 2025a, 2025b). Similarly, in many of the most successful competitive gaming teams, women are either completely absent or very few (Clement, 2025e; Stuart, 2019).

Only a small number of studies have investigated the realities and specificities of women involved in gaming, and most have been focused on the challenges faced by these players such as toxicity, gendered stereotypes and harassment (Lopez-Fernandez et al., 2019a, Kuss et al., 2022; Rogstad, 2021). Despite these challenges and lack of representation and role models, in recent years, some women have begun to break the “glass ceiling” of competitive gaming by competing in international tournaments (Choi et al., 2019; Schelfhout et al., 2021) and some local initiatives have been developed to promote more inclusivity (Hayday and Collison, 2022, pp. 135–137; Sacco, 2020).

However, in research, these competitive women gamers are not well understood. Indeed, the very little available quantitative data precludes an understanding of these women from a psychological standpoint. Thus, the present study represents an initial attempt to draw a psychological profile of these women who persist in competitive gaming environments and their unique characteristics.

### Women and gaming: the social context

Traditionally, playing videogames has been considered a hobby “created by men for men” (Paaßen et al., 2016), with female characters often hypersexualized or in subordinate roles (Davies et al., 2020; Gestos et al., 2018) and a stereotype of the common gamer as male (Williams et al., 2008, p. 995). However, recent data (*n* = 73,000) suggests that women now represent almost half of the gamer base (45%) when including mobile gaming (Ngoc, 2024). Despite this, women are less likely to identify as “gamers” and are rarely visible in skillful and competitive gaming spheres (Kuss et al., 2022; Ngoc, 2024). When they are, they can be subject to disbelief, scrutiny or backlash from parts of the gaming community (Choi et al., 2019; Schelfhout et al., 2021). A large part of scientific writings on female gamers have been qualitative or case studies that have shed light on their experiences of online sexism and harassment and the associated negative impacts on their gaming experience, involvement, motivation, and performance (Colder Carras et al., 2017; Cote, 2015; Kaye and Pennington, 2016; Kuss et al., 2022; Ruvalcaba et al., 2018; Schelfhout et al., 2021; Shen et al., 2016; Vella et al., 2020; Witkowski, 2018). As mentioned by Robinson (2023), this negative impact on women’s full involvement in gaming is susceptible to create a vicious cycle where they are not perceived to be the norm and thus continue to be marginalized (p. 862). In addition to these qualitative data, a few experimental studies have begun to examine the complex impact of social norms and stereotypes on gaming performance with mixed results (Holl et al., 2024; Kaye and Pennington, 2016; Nolla et al., 2023; Pennington et al., 2018). A variety of factors have been suggested to play a role in limiting women’s participation in gaming, especially in competitive spheres. These include, for example, fewer opportunities to begin their involvement in gaming, less support for this activity from the people in their lives, lower levels of belongingness and self-efficacy related to gaming, and social gender-based education (Darvin et al., 2021; Nolla et al., 2023).

On the other hand, little is known about the women who go on to develop their gaming abilities at high levels of expertise despite these challenges (Choi et al., 2019; Schelfhout et al., 2021). Capitalizing on their strengths, it is likely that in the future, more representation of women in competitive gaming would contribute to minimizing challenges and facilitate their greater involvement in this increasingly popular, lucrative and recognized area of expertise (Morgenroth et al., 2020; Pedraza-Ramirez et al., 2020).

### Women’s gaming patterns

Research with female-only gamer samples have shown that women play a variety of games, both online and offline, and across a variety of platforms (Labrador et al., 2022; Lopez-Fernandez et al., 2019b). Compared to men, women report playing “flash” games more frequently (De Pasquale et al., 2020) and having a stronger preference for “platformer” “brain and skill,” or “casual” games (Laconi et al., 2017; Lange et al., 2021; Rehbein et al., 2016). However, some results suggest that women are as interested in and as likely to play Massively Multiplayer Online Role-Playing (MMORPG) games such as World of Warcraft, and that they enjoy Multiplayer Online Battle Arena (MOBA) and First-Person Shooter (FPS) games as much as men (De Pasquale et al., 2020; Laconi et al., 2017). Overall, this data offers limited evidence to back up the stereotype that women prefer more simple, casual games (Paaßen et al., 2016). Interestingly, the study by Lange et al. (2021) found evidence of a moderate overestimation of gender differences in game genre preferences among their participants.

Regarding gaming involvement and motives, recent studies suggest that women spend less time gaming than men (e.g., Cudo et al., 2022; Laconi et al., 2017; Rehbein et al., 2016). Women have been reported to play mainly for the purpose of “having fun,” passing time and immersing themselves in a virtual world (Labrador et al., 2022; Lopez-Fernandez et al., 2019b). Compared to men, women report a higher desire to build relationships through gaming (Laconi et al., 2017; Yee, 2006). Some evidence of gender-specific relations between motives to play and disordered gaming have also been found, suggesting a unique role of achievement, competition and skill development motives (Laconi et al., 2017; Lopez-Fernandez et al., 2019b).

### Problematic use of videogames

Research has suggested that gaming can be a life-enhancing activity but also an attempt to avoid life’s difficulties and/or psychological symptoms (Banyai et al., 2019; Griffiths, 2019). For a minority of players, this can turn into a full-blown addiction (Stevens et al., 2020). Multiple studies which compared GD in male and female gamers have found lower GD scores in women (Laconi et al., 2017; Li et al., 2023), a lower proportion of women among GD players (Bonnaire and Baptista, 2019) or a link between male gender and GD (Holm et al., 2021). However, one study found no gender difference after controlling for game genre (Elliot et al., 2012), one found higher scores in female gamers (Kahraman and Kaya Yertutanol, 2021). Two recent meta-analyses found evidence of a lower risk of GD (Stevens et al., 2020; Su et al., 2020) with a rate of 2.54% in women compared to 6.31% in men (Stevens et al., 2020). However, these data do not allow to understand the interplay between female gaming, competition, and positive and negative outcomes more broadly.

The little gender-specific data available about risk factors for GD suggest that women with gaming problems tend to be older, more often in a romantic relationship, more often unemployed and more likely to have less than a high school education compared to men with GD (Bonnaire and Baptista, 2019). Distraction from problems has also been shown to predict GD in a women-only sample (Labrador et al., 2022). Other risk factors include game genre (i.e., FPS, MMORPG, or MOBA), psychological symptoms, lower self-esteem, lower prosocial behavior, peer problems, body shape concerns, avatar identification and immersion in gaming experiences (Labrador et al., 2022; Lopez-Fernandez et al., 2019b; Thakur et al., 2023).

In general, female players tend to report higher levels of psychological distress and lower self-esteem compared to male players (Finserås et al., 2022; Laconi et al., 2017), both of which are risk factors for GD (e.g., Bonnaire and Baptista, 2019).

### Women as competitive gamers

Players’ engagement in skillful and competitive gaming is a recent area of scientific research. Of this limited literature, most has investigated eSport and professional gaming, where women are scarce (Faust et al., 2020; Reitman et al., 2020). In fact, most studies on competitive eSports and professional gaming participation and associated consequences report proportions of women between 0 and 10% (Banyai et al., 2019; Jansz and Tanis, 2007; Lee et al., 2020; Maldonado-Murciano et al., 2022). On the other hand, in one study by Tang et al. (2021), women represented 36.9% of eSports consumers when both eSports engagement and viewing were considered simultaneously. Overall, the low representation of women in eSports and competitive gaming bears the question if the very few women who do compete have specific characteristics.

From this data, factors related to competitive gaming include time spent online and being motivated to compete, perfect one’s skills, and socialize (Banyai et al., 2019; Jansz and Tanis, 2007; Martoncik, 2015). Competitive players could have an increased risk of experiencing negative consequences such as depressive symptoms, worse sleep quality and GD (Banyai et al., 2019; Lee et al., 2020; Maldonado-Murciano et al., 2022). On the other hand, very little data is available regarding motives, play styles and consequences experienced by female competitive gamers. The few available studies suggest that these women spend less time playing and viewing eSports than men but share the same genre preferences (with the exception of FPS, preferred by men; Tang et al., 2021) and report a higher motivation for achievement (Kordyaka et al., 2023) than competitive male gamers. Additionally, compared to non-competitive female gamers, competitive women were less likely to underestimate their own skill when playing against a “male” artificial intelligence in an experimental study (Vermeulen et al., 2014). Relatedly, some research has begun to examine the impact of “stereotype threat” on the gaming performance and self-perceptions of female players (Holl et al., 2024; Kaye and Pennington, 2016; Pennington et al., 2018). This concept refers to the possibility for cues associated with common social stereotypes (e.g., a reminder of the idea that women are less competent in gaming than men) to negatively impact members of the stereotyped group (Kaye and Pennington, 2016). Although this impact seems unclear in the context of casual forms of gaming, Pennington et al. (2018, p. 12) have suggested that it could impact women during more competitive play, for example due to the higher level of performance pressure, the presence of harassment, and/or the lower representation of women. On the other hand, self-identifying as a gamer could play a favorable role on performance (Holl et al., 2024), and perhaps this could help counterbalance some of the impact of stereotype threat.

However, to our knowledge, no quantitative study to date has been conducted to draw a portrait of competitive female gamers and psychological aspects such as their motives to play, mental health and gaming habits. Obtaining more insight into the specific realities of highly skilled female gamers may offer avenues to promote more inclusiveness of competitive and expert play, in addition to highlighting possible specificities essential to the promotion of their mental wellbeing and the prevention of gaming-related harm.

Thus, the current study aimed to provide initial data about self-identified female expert videogame players, including their sociodemographic and gaming characteristics, motives to play, and mental health. To highlight the characteristics that set them apart as experts while also taking into account the specific reality of female gaming as a reference point, they will be compared to a group of female non-expert, or casual, gamers. A second objective was to identify the factors that predict being a female expert gamer.

## Materials and methods

### Participants and procedure

Our sample was recruited via Facebook, Reddit, and Snapchat publicities and Discord postings from June to October 2020, during the COVID-19 pandemic. The survey was hosted on Qualtrics, preceded by an informed consent form. Apart from the selection criteria (18+, play for 1+ hour per week) and the main game played, no other question was mandatory. After completing the survey, participants were able to enter their email address for a chance to win a US$20 or a US$50 Amazon.com gift card, and/or to be contacted for future studies. From the total sample (*n* = 1,591), only the women who participated (*n* = 128) were examined in the current paper. This study was approved by the institutional ethics review board, Comité d’éthique de la recherche pour les projets étudiants impliquant des êtres humains of the Faculty of Humanities of the Université du Québec à Montréal (CERPE-FSH #4102, 22-06-2020).

### Measures

#### Sociodemographic characteristics

Self-reported information about participants’ sociodemographic characteristics was collected. This included age, relationship status, occupational status, country of residence, and having children at charge.

#### Gaming experience and competence

*Gaming competence.* Players were asked about their subjective level of competence in videogames. Response options were “Beginner,” “Intermediate,” “Expert,” and “Professional.” This variable was inspired from previous studies (Quick et al., 2012; Nagygyörgy et al., 2013) and was then dichotomized to form an “expert” (expert/professional competence) and a “casual” (beginner/intermediate) group.

*Professional gaming and eSports participation.* Players were asked if they played videogames for a living and if they were part of an e-sports team (yes/no).

*Weekly time played.* Players were invited to report their average weekly time spent on their main game (game played for the most hours) and on other games, in hours.

*Years of experience.* Players were asked what year they started playing their main game. Options ranged from 2004 (creation of World of Warcraft) to 2020 (year of the study). Years of experience were then calculated (2020—response).

*Gaming disorder (GD).* Level of GD experienced during the past 12 months was measured using the 10-item *Internet Gaming Disorder Test* (IGDT-10; Kiraly et al., 2019). This instrument uses the DSM-5 (American Psychiatric Association, 2013) proposed criteria for GD, of which the ninth criterion (consequences) is divided into 2 (impact on relationships; impact on work/school) and then recombined. Answers are on a 3-point scale (never/sometimes/often) and are then dichotomized. A minimum of 5/9 symptoms indicates a risk of GD (Kiraly et al., 2019). This scale has shown good psychometric properties in various samples of videogame players (e.g., Kiraly et al., 2019; Laconi et al., 2017; Männikkö et al., 2019).

#### Motivational and psychological aspects

*Motives to play.* Motives to play videogames were measured using Demetrovics et al. (2011) *Motives for Online Gaming Questionnaire* (MOGQ). This scale contains 27 items that measure 7 motivations (social, escape, competition, coping, skill development, fantasy, recreation). Response options range from 1 (*Almost never/Never*) to 5 (*Almost always/Always*). This questionnaire has shown good psychometric properties across many samples of videogame players (e.g., Kim et al., 2016a; Laconi et al., 2017).

*Self-esteem.* Self-esteem was measured using the *Single-Item Self-Esteem Scale* (SISE; Robins et al., 2001), *I have high self-esteem*, measured on a 5-point Likert scale (*1 = not very true of me, 5 = very true of me*). It has shown good psychometric properties, including strong convergent and criterion validity (Robins et al., 2001).

*Impulsivity*. Dysfunctional impulsivity was measured using the *Functional and Dysfunctional Impulsivity scale* (Dickman, 1990). The *Dysfunctional Impulsivity* facet contains 12 dichotomic (true/false) items with a total score of 0–12. This scale has demonstrated good psychometric properties, including with samples of smartphone and videogame users (Kim et al., 2016b; Wang et al., 2018).

*Psychological distress*. General psychological distress was measured using the 10-item *Kessler Psychological Distress Scale* (K10; Andrews and Slade, 2001; Kessler et al., 2002). This instrument uses a 5-point Likert-type scale, with total scores between 10 and 50. Clinical thresholds help identify mild (20–24), moderate (25–29), or severe (30+) distress (Vasiliadis et al., 2015). This scale has shown good psychometric properties, including with gamer samples (Pearcy et al., 2017).

*Mental health diagnosis.* Participants were invited to indicate if they ever received a diagnosis for a mental health disorder (e.g., anxiety, depression).

*Wellbeing.* Wellbeing was measured using the *Mental Health Continuum Short Form* (MHC-SF; Keyes, 2009). This 14-item instrument includes three items that measure emotional wellbeing, six items for psychological wellbeing and 5 for social wellbeing experienced in the past month. Participants are invited to respond using a six-point scale (1 = *Never*, 6 = *Every day*). This scale has shown good psychometric properties across various samples (Lamers et al., 2011; Santini et al., 2020).

### Statistical analysis

To provide an overall portrait of expert female gamers, and to contrast them with casuals, a series of independent-samples t-test analyses were conducted on continuous gameplay-related and psychological variables. Effect sizes were calculated using Cohen’s *d* statistic. Chi-square analyses were conducted for categorical sociodemographic and gameplay-related variables. Effect size was calculated using the Phi-index for two-level variables and Cramer’s V for variables with more than two levels. Finally, a backward logistic regression using the likelihood ratio method allowed to identify the best predictors of being an expert gamer among the significant variables of the chi-square and t-test analyses. All analyses were conducted using SPSS Statistics version 29.

#### Ethics

The study procedures were carried out in accordance with the Declaration of Helsinki. This study was approved by the Comité d’éthique de la recherche pour les projets étudiants impliquant des êtres humains of the Faculty of Humanities of the Université du Québec à Montréal (CERPE-FSH #4102, 06-22-2020) Institutional Review Board. All participants were informed about the study and all provided informed consent.

## Results

### Descriptive statistics

From the total number of women (*n* = 128), 43 were classified as “Expert” and 85 as “Casual” players. Sociodemographic characteristics and group comparisons are shown in Table 1. The average age was 26.50 (*SD* = 8.30). Expert gamers were older than casuals, *t*(126) = 2.161, *p* = 0.03. About three out of four women (74.2%) were in a relationship or married and most (79.7%) were working and/or studying at the time of the study. A total of 38 countries were represented, the most frequent being the United States of America (*n* = 29) and Canada (*n* = 28). Experts were especially likely to live in North America, *χ^2^*(1) = 4.647, *p* = 0.03. Most participants (83.6%) did not have children at charge.

**Table 1:** Sociodemographic characteristics.

Variables	Experts (*n* = 43)	Casuals (*n* = 85)	Total sample (*n* = 128)	*p*	Effect size (Cohen’s *d*/Φ)
**Age**	**28.70 (8.20)**	**25.39 (8.18)**	**26.50 (8.30)**	**0.033**	**0.40**
Relationship status (single)	23.3%	27.1%	25.8%	0.642	
Occupation (is working and/or studying)	83.7%	77.6%	79.7%	0.420	
**Country of residence (North America)**	**61.9%**	**41.5%**	**48.4%**	**0.031**	**0.19**
Children at charge (yes)	20.9%	14.1%	16.4%	0.326	

Results in bold reached statistical significance.

### Motives to play

Motives to play and group comparisons are shown in Table 2. Experts were found to have a higher social motive for gaming than casuals, *t*(126) = 2.746, *p* = 0.007. Experts also had higher competition motive, *t*(126) = 2.746, *p* = 0.004, and a higher skill development motive, *t*(126) = 2.591, *p* = 0.01. Lastly, expert players reported higher recreation motive, *t*(115.709) = 3.436, *p* < 0.001.

**Table 2:** Motivational and psychological characteristics.

Variables	Experts (*n* = 43)	Casuals (*n* = 85)	Total sample (*n* = 128)	*p*	Effect size (Cohen’s *d*/Φ)
Motives to play					
**Social**	**12.81 (3.95)**	**10.87 (3.70)**	**11.52 (3.88)**	**0.007**	**0.51**
Escape	14.14 (5.23)	14.27 (4.46)	14.23 (4.71)	0.88	
**Competition**	**12.81 (5.05)**	**10.34 (4.30)**	**11.17 (4.69)**	**0.004**	**0.54**
Coping	14.09 (4.27)	13.14 (3.47)	13.46 (3.77)	0.18	
**Skill development**	**14.14 (4.64)**	**11.93 (4.52)**	**12.67 (4.66)**	**0.01**	**0.49**
Fantasy	11.95 (5.01)	11.75 (5.23)	11.82 (5.14)	0.84	
**Recreation**	**14.26 (1.45)**	**13.15 (2.15)**	**13.52 (2.00)**	**< 0.001**	**0.57**
Psychological characteristics					
**Self-esteem**	**2.98 (1.44)**	**2.32 (1.18)**	**2.54 (1.30)**	**0.006**	**0.52**
Impulsivity	2.81 (2.99)	2.81 (2.88)	2.81 (2.90)	1.00	
Psychological distress (moderate/severe)	51.2%	64.7%	60.2%	0.139	
Mental health diagnosis	37.6%	46.5%	40.6%	0.348	
**Wellbeing**	**50.58 (16.13)**	**42.34 (15.44)**	**45.11 (16.10)**	**0.006**	**0.53**

Results in bold reached statistical significance.

### Psychological characteristics

Psychological characteristics and group comparisons are shown in Table 2. Expert gamers exhibited higher levels of self-esteem compared to casuals, *t*(126) = 2.772, *p* = 0.006. Experts also reported higher levels of wellbeing, *t*(126) = 2.809, *p* = 0.003.

### Gaming experience

Gaming experience characteristics and group comparisons are shown in Table 3. The weekly time dedicated to the primary game was higher among expert gamers compared to casuals, *t*(126) = 3.209, *p* = 0.002. Additionally, years of experience were higher for experts, *t*(126) = 3.489, *p* < 0.001. The mean GD score was 2.48, with 17.2% of potential GD and no significant difference between groups.

**Table 3:** Gaming experience.

Variables	Experts (*n* = 43)	Casuals (*n* = 85)	Total sample (*n* = 128)	*p*	Effect size (Cohen’s *d*/Φ)
**Weekly time (main game)**	**29.09 (19.16)**	**19.95 (12.80)**	**23.02 (15.77)**	**0.002**	**0.60**
Weekly time (other games)	11.93 (13.06)	11.46 (13.75)	11.62 (13.47)	0.853	
**Years of experience**	**8.81 (4.68)**	**5.72 (4.77)**	**6.76 (4.95)**	**<0.001**	**0.65**
Professional gaming (yes)	3 (7.0%)	3 (3.5%)	6 (4.7%)	0.383	
Esports team (yes)	0 (0.0%)	1 (1.2%)	1 (0.8%)	0.475	
Gaming disorder (yes)	7 (16.3%)	15 (17.6%)	22 (17.2%)	0.846	
Game genre					
Battle Royale	5 (11.6%)	6 (7.1%)	11 (8.7%)	**0.009**	**0.30**
**MMORPG**	**26 (60.5%)**	**29 (34.5%)**	**55 (43.3%)**		
**MOBA**	**6 (14.0%)**	**34 (40.5%)**	**40 (31.5%)**		
Other genre	6 (14.0%)	15 (17.9%)	21 (16.5%)		

Results in bold reached statistical significance.

### Game genre

Type of main game and comparisons are shown in Table 3. A significant difference was highlighted, *χ^2^*(3) = 11.694, *p* = 0.009, with a small to moderate effect size (Cramer’s *V* = 0.30). Specifically, experts demonstrated more engagement in MMORPG games. Conversely, casuals were more inclined towards MOBA games.

### Results of logistic regression analysis

Results of the logistic regression predicting group (expert versus casual) membership are shown in Table 4. Out of the 128 women, 5 had missing data. Thus, the final sample of the regression included 42 experts and 81 casuals. The final model was estimated to explain 40.6% of the variance in group membership (Nagelkerke R square). The Hosmer and Lemeshow test was nonsignificant (*χ^2^*(8) = 12.938, *p* = 0.114), suggesting that the model fit the data well. The final model was better at predicting casual (88.9%) than expert (47.6%) group membership (overall accuracy = 74.8%). The probability of being an expert gamer increased for each hour and year spent on the main game and each point on the competition motive subscale. Additionally, self-esteem had the largest positive relation with being an expert, while playing a MOBA as the main game decreased the odds of being an expert by 77%.

**Table 4:** Results of logistic regression analysis (final model; *n* = 123).

Variable	Exp(B)	*p*	95% CI
Weekly time—main game	1.053	0.006	1.015–1.092
Years of experience—main game	1.124	0.017	1.021–1.237
Competition motive	1.162	0.004	1.048–1.287
Self-esteem	1.552	0.022	1.066–2.258
MOBA main game genre (yes)	0.232	0.011	0.076–0.711

## Discussion

The goal of the present study was to provide an initial portrait of self-identified female expert gamers and to compare them with non-experts, to highlight their specific sociodemographic, gameplay, and psychological characteristics. In addition, the present study aimed to highlight predictors of expert group membership. These women reported playing videogame genres (Battle Royale, MMORPGs and MOBAs) with important social and competitive components, which could be considered to present a risk for GD but also a potential buffer against loneliness in the context of the COVID-19 pandemic (Männikkö et al., 2019; Nebel and Ninaus, 2022). Compared to previous studies, even our casual group reported spending high amounts of weekly time gaming (Cudo et al., 2022; Laconi et al., 2017). However, having put many hours for almost 6 years into their main game, they still reported a low level of gaming competence. This suggests that other factors are involved in estimating their skill level. These could include their level of involvement in gaming activities socially recognized as skillful versus in supporting roles, their exposure to gender stereotypes during play, their self-identification as gamers or others’ recognition of their gaming skills (Davies et al., 2020; Gestos et al., 2018; Holl et al., 2024; McLean and Griffiths, 2018; Ratan et al., 2015).

The fact that expert female gamers were older than casuals had not, to our knowledge, been reported elsewhere. These women were also more likely to live in North America and to play an MMORPG as their main game. The most popular MMORPG, World of Warcraft, originated in the United States, was first launched in North America, Australia and New Zealand^1^ and is still highly popular in North America.^2^ It is possible that the demographic of female experts could have been influenced by these trends. In addition, some recent results suggest that female gamers now play MMORPGs just as frequently as male gamers (De Pasquale et al., 2020). Thus, it is also possible that women who play MMORPGs are more likely to be socially recognized as “true gamers” and/or less likely to face harassment and/or discrimination as their presence in MMORPGs becomes more normalized (Morgenroth et al., 2020). In turn, this could increase their game involvement, promoting their skill development and social participation (Cote, 2015; Kuss et al., 2022; Vella et al., 2020). On the other hand, it is also possible that MOBA games, such as League of Legends, could attract a larger diversity of players including casuals. This would help explain why casuals from our sample were younger on average and relatively more likely to play MOBA games. The fact that the proportion of Battle Royale players did not significantly differ between groups is surprising given that Fortnite tends to attract younger players (Clement, 2023a, 2023b). However, the number of female Battle Royale players in our sample may simply be too small to detect a statistical difference.

Interestingly, time spent playing videogames other than the main one and GD did not differ between groups, which contrasts with previous research which related GD risk to professional gaming (Maldonado-Murciano et al., 2022). Of note however, both groups reported a high prevalence of GD along with high amounts of time spent playing videogames (Laconi et al., 2017; Maldonado-Murciano et al., 2022; Stevens et al., 2020). Therefore, this result warrants replication across different samples. Of note, while the rates of moderate-to-severe psychological distress did not differ between groups, it was still quite high, which can be partly explained by the social context of the COVID-19 pandemic (Patel et al., 2022).

Compared to casuals, experts were shown to report higher levels of social, competition, skill development, and recreation motives. These results highlight what is unique in women with high gaming competence, which, to our knowledge, had not been documented before. Previous research had found that male players reported higher social, competition, skill development, fantasy, and recreation motives in comparison to female gamers (Laconi et al., 2017). In addition, compared with casuals, competitive gamers tend to report a higher desire to socialize, compete and develop their skills (Banyai et al., 2019; Jansz and Tanis, 2007). Since men are generally more involved in gaming, especially in competitive play, where aspects of competition, skill and collaboration are central, some of the previously found gender disparities could perhaps be attributable to this greater involvement in skillful play rather than to a real gender difference, although this would need to be verified (Banyai et al., 2019; Cudo et al., 2020; Laconi et al., 2017).

In addition to their motivational specificities, expert female gamers were also found to report higher self-esteem and wellbeing. Given the obstacles faced by women involved in gaming (Kuss et al., 2022), it is possible that various personal strengths could better equip experts to maintain a high involvement in competitive spheres over the years (Cote, 2015; Vermeulen et al., 2014). This includes, as highlighted here, higher self-esteem and wellbeing, but could also encompass personality variables such as extroversion, mental toughness and emotional stability, as well as environmental factors like in-game successes, in-game social recognition and inclusion, and social support outside of the game environment (Abarbanel and Bernhard, 2012; Darvin et al., 2021; Vollstädt-Klein et al., 2010). Of note, being socially recognized for one’s high performance and success in an environment where these are central could create rewarding and self-esteem promoting experiences for these women (Faust et al., 2020; Reitman et al., 2020). Unfortunately, successes by especially high performing female gamers have sometimes generated negative reactions, disbelief and scrutiny from eSports communities rather than (or alongside) positive recognition (Choi et al., 2019; Schelfhout et al., 2021). However, following recent initiatives, more inclusivity could, over time, change social perceptions of what constitutes a gamer, promoting better integration and recognition of women experts (Morgenroth et al., 2020).

Results of the backward logistic regression suggested that the most reliable predictors of being an expert female gamer were *not* playing a MOBA game for the most time, spending more time playing one’s main game, having higher self-esteem, being motivated to compete in videogames, and having more years of experience on one’s main game. The fact that playing a MOBA as the main game was associated with being a casual player is surprising as this genre is frequently associated with eSports (Pedraza-Ramirez et al., 2020). Further investigation could help determine if this could be due to a bigger gender disparity in participation (social explanation) and/or to specific structural characteristics of MOBAs. The presence of time spent playing and years of experience among the best predictors makes logical sense in that gaming success is usually seen as requiring time and practice (Faust et al., 2020). However, recent mixed results regarding the effects of practice on expertise support that it is not the only factor at play (Pedraza-Ramirez et al., 2020). Indeed, in the model presented here, it is the combination of time and years spent playing with high desire to compete, self-esteem and being involved in specific game genres that allowed to reliably predict group membership. The fact that competition and self-esteem were among these predictors suggests that in contrast to results obtained with gender comparisons which consider all women as a single group (e.g., Laconi et al., 2017), female expert gamers can be highly competitive and report normal levels of self-esteem.

## Conclusion

The current study allowed to provide the very first portrait of women who perceive themselves as expert gamers. Results allowed to highlight important heterogeneity among women gamers, including specific motivations, psychological characteristics and gaming experience of women experts. These results provide an encouraging outlook for women who sustain their highly involved gaming practices despite social obstacles of the traditionally masculine gaming world, pointing to the importance of factors such as representation and social integration. Despite these important advancements, this study has some limitations that are important to consider. Firstly, our subsample of women is non-representative, as it is relatively small and comprised of self-selected participants recruited from social media, with an overrepresentation of women residing in North America. This may impact the external validity of our findings and limits their generalizability to the broader world population of female gamers. Thus, results should be replicated with other, and if possible, bigger, samples of female gamers. Secondly, our data is self-reported and thus subject to reporting biases. This includes, for example, biases related to social desirability and over- or under-reporting. In addition, while stereotype threat and gamer identity were not examined in our study, it may have had an impact on participants’ self-assessments (Holl et al., 2024; Kaye and Pennington, 2016). Thirdly, while self-reported expertise allows insight into the reality of competitive female gamers, it cannot be equated with objective competence or actual engagement in competitive gaming activities. Moreover, while recoding self-reported expertise into a binary classification was essential in allowing to draw comparisons between casual and expert gamers, this may not be an accurate reflection of the nuanced gradations of this subjective variable. Finally, combining all female gamers into a single group and using only a quantitative design allowed to provide a substantial contribution to current scientific knowledge of female gamers with good statistical power. However, this does not allow for an intersectional analysis (e.g., race/ethnicity) or qualitative insights as to the contributors to group membership. Thus, future studies that would delve deeper into female eSports players (although their numbers seem very limited) or women with certain objective markers of competence would complement this one well. As for future directions, further comparing gaming areas where women are more (or less) represented while going more in-depth about the mechanisms at play may provide insight into possible strategies to promote their integration into competitive gaming spheres. In particular, the impact of gaming on self-esteem and vice-versa would be a relevant area to explore.

## Data Availability

The datasets presented in this article are not readily available to preserve individual’s privacy, as agreed with our institutional ethics committee. At the time of the study (2020), participants agreed to their data only being used for the project presented here. Requests to access the datasets should be directed to Roxanne Hébert-Ratté, hebert-ratte.roxanne@courrier.uqam.ca.

## Footnotes

^1^ https://wowpedia.fandom.com/wiki/Timeline_(World_of_Warcraft)

^2^ https://worldofwarcraft.fandom.com/et/wiki/WoW_population_by_country
